# Development of a Database of LanguaL^TM^ and FoodEx2 Codes of 50 Ready-to-Eat Products

**DOI:** 10.3390/nu16081151

**Published:** 2024-04-12

**Authors:** Alessandra Durazzo, Tommaso D’Andrea, Paolo Gabrielli, Niccolò Pilla, Altero Aguzzi, Massimo Lucarini, Gianni Sagratini

**Affiliations:** 1 CREA-Research Centre for Food and Nutrition, Via Ardeatina 546, 00178 Rome, Italy; 2 Chemistry Interdisciplinary Project (ChIP), School of Pharmacy, University of Camerino, Via Madonna delle Carceri, 62032 Camerino, Italy; 3 Università di Torino, Via Verdi, 8, 10124 Turin, Italy; 4 Università Campus Biomedico di Roma, Via Álvaro del Portillo, 21, 00128 Rome, Italy

**Keywords:** coding procedure, food classification, food description, LanguaL^TM^, FoodEx2, database, data sharing, ready-to-eat (RTE) products, ready-to-heat (RTH) products, food preparations, composite dishes

## Abstract

Ready-to-eat (RTE) and ready-to-heat (RTH) dishes are food items that help save time, physical energy, and mental effort in all food-related activities. Convenience of use, variability of supply, and adaptability to different consumption occasions have led to an increase of acceptance among consumers through the years. Specialized databases can help in this context, where food composition databases can provide information and data to create sustainable nutritional models by reducing the now growing number of chronic diseases. This paper aims at developing a database of LanguaL^TM^ and FoodEx2 codes of 50 food preparations and ready-to-eat dishes designed for consumption outside the home. LanguaL^TM^, as well as FoodEx2, are classification and description systems for indexing, in the sense of a systematic description, of foods based on a hierarchical model (parent–child relationship), thus facilitating the international exchange of data on food composition, consumption, assessing chronic and/or acute exposure to a certain agent, and not least the assessment of nutrient intake. The database, here presented, consists of the codes of fifty ready-to-eat products present on the market in Italy, obtained by using the two mostly commonly used and widely recognized coding systems: LanguaL^TM^ and FoodEx2. This database represents a tool and a guideline for other compilers and users to apply coding systems to ready-to-eat products. Moreover, it can be represented a resource for several applications, such as nutritional cards, nutritional facts, food labels, or booklet and brochures for promotion of food products, to be used at health and food nutrition interface, useful for consumers, dieticians, and food producers.

## 1. Introduction

RTE meals are food items that, as compared to meals made from raw ingredients, help save time, physical energy, and mental effort in all food-related activities [1,2,3]. Convenience of use, variability of supply, and adaptability to different consumption occasions have led to an increase of acceptance among consumers through the years. Their success comes from the fact that these products are designed and developed so that they are convenient to consume in the context for which they are supposed to be consumed, such as a quick lunch break or a home meal whose preparation does not take much time.

Lifestyles have undergone changes propelled by the increasingly hectic pace of daily life, necessitating a need for reorganization. Factors such as a declining birth rate, a rising number of single individuals, the expansion of female employment, longer daily work hours, and increased distances between workplaces and homes have collectively contributed to a reduction in available time for meals and a transformation in the structure of meals themselves.

Currently, consumers are becoming more conscious, largely due to the widespread dissemination of active information within the realm of food. Ready-to-eat products are well suited in a situation where there is less and less time available to be able to devote to meals and people want to comfortably eat a meal out in work environments.

IV (RTE ready-to-eat) and V gamma (RTH ready-to-heat) products [3] require less time for preparation and minimal strain than less processed products belonging to I and II categories. They both go to satisfy the requirement for a quick lunch in a work situation, pandering to practicality of use in a fast-paced context such as a big-city lifestyle, while also staying true to health.

At the same time, it is important to have databases that include data on cooked foods, processed foods, and food preparations (recipes) [4,5,6], which are both harmonized and standardized [7,8]. Nowadays, it is important to evaluate the food as it is consumed. Most products are consumed after processing or cooking. Often, they are consumed as food preparations and recipes.

Over the years, the studies in the literature have been focused on individual products, while only a few studies analyze the nutritional composition of food preparations and recipes. For that reason, it is important to develop a code database for all the categories of meals. In this context, it is important to have data collected in a standardized and harmonized manner. Food, nutrition, and health are concepts that are closely related to each other. Over the last decade, there has been an increase in the number of studies and research on the connection between these different sectors. Specialized food databases can provide information and data for the creation of sustainable nutritional models to reduce the now growing number of chronic diseases [8,9,10,11,12].

Data exchange is streamlined through standardized data collection methods, promoting easier comparison. Harmonized data exchange and cross-country collaborations are enhanced when databases use a common language, fostering greater autonomy in the process [8,12,13,14].

Databases not only allow insight into human nutrition and public health, but also prove to be useful and necessary systems for professionals in the sector: nutritionists, dieticians, food developers, and researchers, for several applications and fields: diet assessment, exposure studies, food labeling, epidemiological studies, clinical research, nutrition education, and support for food industries for food labeling.

Data should comply with FAIR principles (findability, accessibility, interoperability, and reusability). With new emerging technologies, infrastructures are emerging that can make this process adequate [11,12,15].

Designing and developing a database requires an adequate classification and description of foods.

LanguaL^TM^ and FoodEx2 are the two systems with excellent international reputations [16] that are able to describe and classify food according to those intrinsic characteristics of the product itself. LanguaL^TM^ and FoodEx2 represent the main classification and description systems, as they are well developed, widely used, and recognized at European and international levels; their common use represents the future direction, in the perspective of an automated interchangeability system.

LanguaL™ [17,18] is a multi-faceted food classification system that allows systematic food description using a diversified set of facet terms and different levels of detail. FoodEx2 [19,20,21,22,23,24,25,26] represents a system of flexible combinations of classifications and descriptions based on a hierarchical system for different food safety-related domains. The FoodEx2 system is now at its Revision 2 version. The previous version was FoodEx2 (Revision 1) [27] and the original system was FoodEx1 [28,29].

In detail, LanguaL™ [17,18] is a controlled vocabulary for systematic food description and a multilingual thesaural system that uses facets to describe food properties. It utilizes the thesaurus, a standardized language for describing foods, specifically for classifying food products for information retrieval. Food can be described by a set of standard and controlled terms (taken from facets) describing the nutritional and/or hygienic quality of a food, i.e., the biological origin, the cooking and conservation methods, and technological treatments; the identification of meaningful characteristics is given by the use of descriptor codes and the arrangement of facet terms in a hierarchic structure. Hierarchical relationships allow for the aggregation of numerical values when the food vocabulary is used in compiling data.

The FoodEx2 [19,20,21,22,23,24,25,26] is a standardized food classification and description system developed by the European Food Safety Authority (EFSA). It consists of a lot of individual food items aggregated into a large set of 21 food groups clearly defined and organized in a hierarchical relationship that represent the minimum recommended level for coding during data collection. Descriptors, grouped in 32 “facets”, represent characteristics of foods from different points of view, describing food properties and aspects from various perspectives, i.e., part nature, ingredient, packaging material, production method, qualitative information, process, and target consumer. F01 indicates Source, F03 Physical state, F18 Packaging format, F19 Packaging material, F21 Production method, and F28 Process: these represent some examples.

There are few studies that carry out LanguaL^TM^ and FoodEx2 coding systems applications on complex matrices such as ready meals. As anticipated, the consumption of the meals outside the home is an important aspect to evaluate in an increasingly frequent phenomenon. The ready-to-eat products are gastronomic preparations made by combining more ingredients and processes together, for consumption outside the home. The coding of these foods could be very challenging due to the complexity of the compositions and the multitude of ingredients.

In this context, this paper aims at developing a database of LanguaL^TM^ and FoodEx2 codes of 50 food preparations and ready-to-eat dishes designed for consumption outside the home.

This study is suggested as a novelty approach, serving as a tool and guide for other compilers and users for the application of coding systems (LanguaL^TM^ and FoodEx2) for ready-to-eat meals.

A database of LanguaL^TM^ and FoodEx2 codes of 50 ready-to-eat products was developed. The database, here presented, consists of the codes of fifty ready-to-eat products present on the market in Italy. It is a resource for several applications at the interface of nutrients and health for dieticians, consumers, and food producers.

## 2. Materials and Methods

### 2.1. Selection of Products

The food products screened for this work have been identified and selected by thinking about a quick and ready solution not only for the lunch break in working environments, but also at home, evaluating those factors such as the lack of time together with the increasing distance between the place of work and home. At the same time, another criterion has been assessed, and that is the need for a clear and complete label covering aspects such as the ingredients used, the method of cooking, and the method of preservation.

Considering these aspects and criteria, the study focused on products placed under the macro-category of IV gamma and V gamma, also referred to as ready-to-eat and ready-to-heat meals. These products are defined as time saving, capable of combining convenience and consumption. The term gamma means the degree of processing of the product. Salads, cut and packaged vegetables, and ready-to-use peeled fruit are some of the countless products that dot the IV gamma category. They are fresh and minimally processed and ready-to-use foods. They are frequently washed, cut, and packaged in modified atmosphere packaging (MAP).

Products in the V gamma category have undergone an extra degree of processing than their counterparts. They are precooked to be immediately ready for use or ready for use after brief heating, but not frozen (frozen foods belong to the III category). The products in the V gamma category are packaged as pre-prepared meals and are often already divided into portions. The expected shelf-life of IV gamma products is about 7–21 days. V gamma products, with only one more degree of processing, have a minimum shelf-life of 6 days and a maximum shelf-life of 6 months, according to the type of treatment applied.

The analysis and the choice of products was carried out by going in person to various retail outlets in Rome, as well as consulting the relevant home shopping websites of the same stores. Online catalogues of other companies—not large-scale retailers—that sell food and ready meals were also consulted. The selected products are therefore available both at physical stores and online.

### 2.2. Coding Procedure

LanguaL^TM^ [17,18] and FoodEx2 [19,20,21,22,23,24,25,26] coding software were chosen for the coding of these products because they are recognized by different European and international bodies for their widespread use in food classification and description.

The coding procedure was performed by a qualified compiler that constantly follows updates of LanguaL^TM^ and FoodEx2 systems. The same compiler was for both encoding systems. A double-check was made by another qualified compiler. The latest versions of the LanguaL^TM^ system [17,18] and FoodEx2, Revision 2 [19,20,21,22,23,24,25,26] were used.

LanguaL^TM^ was established for the purpose of sharing data exchange among national food composition databases. LanguaL^TM^ exploits the LanguaL^TM^ Food Product Indexer Software. The code for LanguaL^TM^ is set a priori. The facets that best describe the food are chosen, and the same pattern should be maintained for all food items. The descriptors are explicated and structured.

FoodEx2, Revision 2, was developed as a food description and classification system specifically for risk-assessment studies and uses EFSA’s Catalogue Browser.

FoodEx2 also uses, unlike LanguaL^TM^, implicit descriptors in order to reduce the code length. This allows the compiler to consider each food item as it best sees fit without the need to keep track of a coding scheme to maintain for each food item.

The correct application of classification and description systems is based on official protocols, continuous updating, and multi-disciplinary skills.

## 3. Results and Discussion

A database of LanguaL^TM^ and FoodEx2 codes of 50 ready-to-eat products was developed and reported in the Supplementary Material; particularly in Table S1 (see Supplementary Materials), 50 ready-to-eat products have been reported; in Table S2 (see Supplementary Materials), the LanguaL^TM^ codes of the 50 products have been reported; and in Table S3 (see Supplementary Materials), the FoodEx2 codes of the 50 products have been reported.

### 3.1. Application of LanguaL^TM^

Table S2 (see Supplementary Materials) reports the LanguaL^TM^ codes of the 50 products.

The following facet scheme has been set and configured for LanguaL^TM^ encoding: [A] = PRODUCT TYPE; [B] = FOOD SOURCE; [C] = PART OF PLANT OR ANIMAL; [E] = PHYSICAL STATE, SHAPE OR FORM; [F] = EXTENT OF HEAT TREATMENT; [G] = COOKING METHOD; [H] = TREATMENT APPLIED; [J] = PRESERVATION METHOD; [M] = CONTAINER OR WRAPPING; [P] = CONSUMER GROUP/DIETARY USE/LABEL CLAIM. The descriptors considered for facet [A] were always chosen from those within “EUROFIR FOOD CLASSIFICATION [A0777]” and “PRODUCT TYPE, U.S. CODE OF FEDERAL REGULATIONS, TITLE 21 [A1270]” as the second alternative.

Facet [B] concerns the food source, defining the individual plant or animal from which the product or its major ingredient is derived.

Facet [C], part of plant or animal, allows us to choose from which part of the plant or animal the product or its main ingredient is derived.

Facet [E] applies aspects such as physical state, shape, or form. The physical state of the food product is categorized as a liquid, semiliquid, semisolid, or solid. Solid food products are further subdivided by shape or form.

Facet [F] is used to evaluate the extent of heat treatment. This is used to broadly characterize a food product based on the extent of heat applied. Heat treatment affects the flavor and textural characteristics of a food and thus consumer preparation time. Heat treatment causes chemical changes and/or reduction of enzyme and of microbial activity and thus affects food safety and shelf-life.

Specifics of preparation are covered by facet [G] = COOKING METHOD and [H] = TREATMENT APPLIED.

The default facet [H] is useful to describe the treatment applied, but it was used by us to describe and classify the ingredients added to the product thanks to the descriptor INGREDIENT ADDED [H0225] and the terms hierarchically cataloged below it.

Facet [M] used descriptors to give information about packaging format and material. The international recycling code, a number code tied to the plastic material [30], was used to specify, if needed, the type of material in the remark field.

The facet [P] = CONSUMER GROUP/DIETARY USE/LABEL CLAIM has always seen the use of one and only one descriptor, used for all fifty products: [P0024] = HUMAN CONSUMER, NO AGE SPECIFICATION.

This scheme has been respected and applied for the coding of all fifty gastronomic proposals analyzed in this work.

We can cluster the items analyzed into the following groups: salads, main courses, side dishes, sandwiches, fruits, and derivatives. This classification is useful for explicitly describing the coding.

#### 3.1.1. Salads

Below are the comments on the coding work done for the category of salads. For FACET [A] related to product type, the descriptors [A0866] = PREPARED SALAD (EUROFIR); [A0208] = SALAD (US CFR) are used for all four products.

The facet [B] descriptor chosen for all products was [B1566] = LEAFY VEGETABLE. The [B] descriptor is used to provide the *food source*, which in this case is vegetable leaves. This general descriptor was chosen because among the vegetable leaves that compose the salad products, there is no significant predominance of one type of leaf over the others present.

The descriptor chosen for facet [C] as well as [E], [F], [G] turns out to be the same for all four products categorized as salads.

Descriptor [C] is associated with descriptor [B], [C0200] = LEAF was chosen as the descriptor to describe the *part of plant or animal* used, and in this case, the leaves are the plant part used.

Facet [E] is useful to assess the *physical state*, *shape*, *or form* of the product. The descriptor used to describe the physical state is consistent among all products of the category: [E0104] = WHOLE AND PIECES is the descriptor chosen according to the fact that the leaves are present in whole form, but they are also cut or not perfectly intact.

Descriptors [F] and [G] indicate the *extent of heat treatment* applied to the product and the *cooking method*, respectively. The entire category was not heat-treated and had no cooking method applied, so the descriptors [F0003] = NOT HEAT-TREATED and [G0003] = COOKING METHOD NOT APPLICABLE were respectively applied.

Facet [H] *treatment applied* employed the descriptor [H0212] = VEGETABLE ADDED, which was used to describe the added ingredients, which in this case are from strictly plant sources. Two products have featured the specific descriptor [H0350] = TOMATO ADDED because they had tomatoes inside. Three out of four products have cheese added, and therefore have the specific descriptor [H0143] = CHEESE ADDED.

Facet [J] is used to describe the *preservation method* of the product, and since they are all fresh products that need the cold chain, the descriptor [J0131] = PRESERVED BY CHILLING was chosen, and this means preservation that must take place at refrigeration temperatures. The facet [M] was used to describe the packaging (*container or wrapping*) of the selected product. [M0124] = PLASTIC TRAY OR PAN, PLASTIC COVER OR WRAPPING was useful in three cases out of four; twice the descriptor [M0124] = PLASTIC TRAY OR PAN, PLASTIC COVER OR WRAPPING was accompanied by the specification for the material inherent in the tray, namely [M0430] = POLYETHYLENE TEREPHTHALATE (PET) CONTAINER when the material of the plastic tray was polyethylene terephthalate, commonly known as PET.

For one type of product, the following descriptors are used: [M0126] = PLASTIC TRAY OR PAN; [M0166] = PLASTIC BAG OR POUCH. Let us now analyze the useful remarks to explicitly state what the use of certain descriptors employed to classify and describe the products clustered as salads actually served.

Since the products were composed of a variable mixture of leafy vegetables, for facet [B], the descriptor [B1566] = LEAFY VEGETABLE was used; it was specified in the remarks of the type of leafy vegetables present as ingredients. Two products are composed of broadleaved endive and rocket; another is composed of a variable mixture of broadleaved endive, rocket, and radicchio; the third is broadleaved endive and rocket; and the last is round red radicchio and lamb’s lettuce.

The descriptors of facet [H] are those with the most remarks. [H0212] = VEGETABLE ADDED has undergone remarks to explain the addition of pitted green olives (twice), pitted black olives, and finally carrots. [H0350] = TOMATO ADDED, on the other hand, underwent remarks twice because it was intended to emphasize that cherry tomatoes were the type added. The remark was also chosen for [H0143] = CHEESE ADDED to specify the type of cheese added, namely Grana Padano PDO in one case and Feta added in the other case. Note how the descriptor [H0143] = CHEESE ADDED has been used three times, and shows only two remarks: this is because, in one case, the label does not specify the added cheese, leaving a generic “flaked cheese” on the label. [H0333] = SEED ADDED was intended to explicitly state that these were walnuts added to the product. Also, unrelated to the facets and descriptors, remarks were useful to emphasize the fact that the salads contain a seasoning kit consisting of salt, extra virgin olive oil, and balsamic vinegar of Modena PGI.

Analyzing the remarks related to the facet [M] concerned the packaging, and comments related to the type of plastic of the container and its format were added in the remarks.

#### 3.1.2. Side Dishes

One of the other product categories is side dishes. Below are the descriptors used for this category.

For the facet [A] related to the type of product, the following descriptors have been used in combination, [A0828] = VEGETABLE DISH and [A0172] = PREPARED FOOD PRODUCT (US CFR), for a total of seventeen times, whereas for the other two items, the combination of descriptors applied was: [A0830] = POTATO DISH (EUROFIR) and [A0106] = PREPARED GRAIN OR STARCH PRODUCT (US CFR).

The descriptor [B1579] = VEGETABLE-PRODUCING PLANT is used three times within this category. Descriptors like [B1458] = EGGPLANT; [B1218] = POTATO; [B1466] = ARTICHOKE; [B1443] = BROCCOLI; [B1552] = CHICORY; [B1420] = SPINACH; [B1227] = CARROT; [B1338] = PEA; [B2632] = CONE PEPPER; [B1456] = PUMPKIN; [B2940] = RED BEET; [B1175] = CHARD; [B1300] = ONION were used in this category.

Facet [C] *part of plant or animal* is related to facet [B]. The most used [C] descriptor, by coding items in this category, was the [C0240] = ROOT, TUBER OR BULB, WITHOUT PEEL. In the remarks field, there is then the reference note with the specification if the part used was a root, a tuber, or a bulb; in all three cases, and that the outer layer is removed, as described.

The second-most frequently used descriptors are [C0200] = LEAF and [C0174] = PART OF PLANT. Specifically, [C0174] = PART OF PLANT has been used in association with the descriptor [B1579] = VEGETABLE-PRODUCING PLANT. The third-most used descriptors belonging to facet [C] are [C0140] = FRUIT, PEEL PRESENT, CORE, PIT OR SEED PRESENT, and [C0151] = HEAD (PLANT). The following descriptors were also used: [C0237] = FLORET OR FLOWER; [C0155] = SEED; [C0162] = HEART (PLANT); [C0229] = FRUIT, PEEL REMOVED, CORE, PIT OR SEED REMOVED; [C0290] = BULB; [C0139] = FRUIT, PEEL PRESENT, CORE, PIT OR SEED REMOVED.

The descriptors belonging to facet [E] *physical state*, *shape*, *or form* used for the *Side dishes* items are four: [E0152] = DIVIDED INTO PIECES (eight times); [E0131] = WHOLE and [E0104] = WHOLE AND PIECES (both four times); [E0137] = SLICED and (three times).

The descriptor [F0014] = FULLY HEAT-TREATED, related to facet [F], is used for all items under *Side dishes* category.

Regarding facet [G] *cooking method*, the descriptors [G0021] = COOKED IN STEAM and [G0015] = BOILED AND DRAINED are mostly used. Other descriptors used are: [G0006] = BROILED OR GRILLED, [G0027] = SAUTEED, [G0005] = BAKED OR ROASTED, [G0018] = BOILED AND UNDRAINED, [G0029] = DEEP-FRIED.

The [H] descriptors are used for indicating other ingredients and specifies added in remark field.

The descriptors used for facet [J] *method of preservation* are only two: [J0131] = PRESERVED BY CHILLING and [J0174] = PRESERVED BY STORAGE IN VACUUM; being that the whole category requires cold chain, [J0131] was chosen for all 19 products. The descriptor [J0174] = PRESERVED BY STORAGE IN VACUUM was used for seven of these products, additionally to the descriptor[J0131], because these products in addition to required storage at refrigeration temperature, they were also stored in a vacuum bag.

For the facet [M] *container or wrapping*, three descriptors were mainly used. The most used one (twelve times) is [M0124] = PLASTIC TRAY OR PAN, PLASTIC COVER OR WRAPPING, which describes those products that present themselves on a plastic tray and with a plastic film as a sealing element. The other descriptor used was [M0166] = PLASTIC BAG OR POUCH with the remark specification that implies that the descriptor was chosen for those products that are wrapped by a plastic bag to create a vacuum as well as for details on packaging material.

#### 3.1.3. Second Courses

For the items under the category of second courses, the following descriptors are used: the descriptor [A0804] = SEAFOOD DISH (EUROFIR) for fish-based products/preparations and the descriptor [A0799] = MEAT DISH (EUROFIR) for meat-based products. Both categories of second courses were classified with the descriptor [A0172] = PREPARED FOOD PRODUCT (US CFR). Respectively, in this category, there are five fish-based products and three meat-based products, totaling eight products (see Supplementary Materials).

Relative to facet [B], the major descriptor used is [B1457] = CHICKEN. All other facet [B] descriptors used are: [B1514] = OCTOPUS; [B1587] = ATLANTIC SALMON; [B3837] = EUROPEAN ANCHOVY; [B1427] = SWORDFISH; [B1059] = SHELLFISH OR CRUSTACEAN; [B1236] = TURKEY (POULTRY).

Recall that facet [B] pertains to the food source and is associated with facet [C], which denotes the parts used. In this case, the parts utilized are from animals. The descriptor that is most commonly used is [C0103] = MEAT PART, followed by [C0268] = SKELETAL MEAT PART, WITHOUT BONE, WITHOUT SKIN; [C0105] = WHOLE ANIMAL, WITH SKIN, FEATHERS OR SCALES, EVISCERATED.

For *physical state*, *shape*, *or form*, the facet descriptors [E] used were: [E0137] = SLICED; [E0131] = WHOLE; [E0104] = WHOLE AND PIECES.

For the *extent of heat treatment*, facet [F], the following descriptors are used: [F0014] = FULLY HEAT-TREATED; [F0003] = NOT HEAT-TREATED; [F0001] = EXTENT OF HEAT TREATMENT NOT KNOWN.

For the facet [G], describing the *cooking method*, the descriptors used are: [G0003] = COOKING METHOD NOT APPLICABLE; [G0006] = BROILED OR GRILLED; [G0029] = DEEP-FRIED; [G0021] = COOKED IN STEAM; [G0005] = BAKED OR ROASTED. For [G0005] = BAKED OR ROASTED in the remarks field, it has been specified that baking occurs at low temperatures.

The facet [H] descriptors used for the items under Second course category are: [H0367] = SALT ADDED; [H0151] = SPICE OR HERB ADDED; [H0347] = SAFFLOWER OR SUNFLOWER OIL ADDED; [H0348] = PARSLEY ADDED; [H0396] = MARINADED; [H0362] = FRUIT JUICE ADDED; [H0172] = SMOKED OR SMOKE-FLAVORED; [H0186] = EGG ADDED; [H0188] = BREADED OR BATTER-COATED; [H0152] = GRAIN ADDED; [H0184] = MILK ADDED; [H0143] = CHEESE ADDED. Specifies are added in remark field as indicated in Supplementary Materials.

Since they are all products that can be consumed immediately or almost immediately, they require the cold chain as a *preservation method*. For facet [J], the descriptor chosen is common to the entire category, and it is [J0131] = PRESERVED BY CHILLING.

To describe *packaging or wrapping*, the descriptors related to facet [M] used were as follows: [M0124] = PLASTIC TRAY OR PAN, PLASTIC COVER OR WRAPPING; [M0430] = POLYETHYLENE TEREPHTHALATE (PET) CONTAINER. For the [M0124] = PLASTIC TRAY OR PAN, PLASTIC COVER OR WRAPPING is specified in the remarks that the packaging is composed of as follows: a plastic tray, type 05 covered by a plastic film, type 07 used as a sealing element.

#### 3.1.4. First Courses

The category of first courses has been subdivided for practical purposes as follows: Pasta; Soups; Cereal Salads.

##### First Courses, Pasta

[A1204] = PASTA DISH (EUROFIR) and [A0220] = PASTA DISH (US CFR) are used for describing the *type of product* for the two products in this category.

For both products, the descriptors [B1079] = DURUM WHEAT; [C0208] = SEED, SKIN REMOVED, GERM REMOVED (ENDOSPERM); [E0134] = SEMISOLID WITH SOLID PIECES, are used for indicating, respectively, the main *food source*, the related *part of plant or animal*, or the *physical state*, *shape*, *or form*.

The descriptor [F0023] = HEAT-TREATED, MULTIPLE COMPONENTS, DIFFERENT DEGREES OF TREATMENT is used to indicate the presence of several different components, each of which may have had a different extent of heat treatment.

The descriptor [G0015] = BOILED AND DRAINED is used for both products for indicating the boiling and draining of pasta. For one product, an additional facet, [G0027] = SAUTEED, has been used.

The [F] descriptors common to both products are: [H0212] = VEGETABLE ADDED; [H0341] = OLIVE OIL ADDED, whereas the other ones used are: [H0180] = FOOD ADDED; [H0151] = SPICE OR HERB ADDED; [H0347] = SAFFLOWER OR SUNFLOWER OIL ADDED; [H0349] = ONION ADDED; [H0367] = SALT ADDED; [H0759] = MEAT PRODUCT ADDED.

In the instance that the descriptor [H0212] = VEGETABLE ADDED was used, the type of vegetables added have been specified in the remarks section; for the descriptor [H0341] = OLIVE OIL ADDED, extra-virgin olive oil has been specified; for the descriptor [H0759] = MEAT PRODUCT ADDED, the type of meat (pork sausage added) has been specified. Then, [H0180] = FOOD ADDED was used to indicate that basil pesto was added to the product.

For the facets [J] *preservation method*; [M] *container or wrapping*, the following descriptors have been used for both items:: [J0131] = PRESERVED BY CHILLING; [M0124] = PLASTIC TRAY OR PAN, PLASTIC COVER OR WRAPPING.The remarks were used to better explicate the type of material of tray and plastic film used as the sealing element. Both trays turned out to be made of plastic material (polypropylene; PP is the abbreviation and 05 is the international recycling code); both tray cover films are made of plastic material (mixed plastic; OTHER; 07 is the international recycling code).

##### First Courses, Soups

The second category related to the first courses was the soups. The following descriptors are used, respectively, for the three products belonging to this subcategory: -[A0865] = SOUP (EUROFIR), [A0243] = SOUP, THIN (US CFR); -[A0828] = VEGETABLE DISH (EUROFIR), [A0180] = SOUP, THICK (US CFR); -[A0832] = PULSE DISH (EUROFIR), [A0172] = PREPARED FOOD PRODUCT (US CFR). The choice of [A0828] = VEGETABLE DISH (EUROFIR) was related to the predominance of vegetables in the product, whereas for the other product the choice of [A0832] = PULSE DISH (EUROFIR) was related to the fact that the product was a pulses (lentils)-based preparation.

The descriptors [B1579] = VEGETABLE-PRODUCING PLANT and related [C0174] = PART OF PLANT are respectively used for indicating the *food source* and *part of plants or animal* for two of these products. For the latest product, [B1268] = LENTIL and related [C0155] = SEED are used.

The following facet [E] descriptors are used for the three products belonging to this category: [E0135] = SEMILIQUID WITH SMOOTH CONSISTENCY, [E0149] = LIQUID, LOW VISCOSITY, WITH SOLID PIECES and [E0104] = WHOLE AND PIECES.

For the *extent of heat treatment*, the facet [F] descriptors were used: [F0014] = FULLY HEAT TREATED and [F0023] = HEAT TREATED, MULTIPLE COMPONENTS, DIFFERENT DEGREES OF TREATMENT.

For all three products, [G0020] = SIMMERED, POACHED OR STEWED is added to indicate simmering as a *cooking method*.

The facet [H] descriptors used are: [H0367] = SALT ADDED, [H0341] = OLIVE OIL ADDED, [H0151] = SPICE OR HERB ADDED, [H0148] = WATER ADDED; [H0180] = FOOD ADDED; [H0323] = BARLEY ADDED; [H0152] = GRAIN ADDED; [H0350] = TOMATO ADDED; [H0212] = VEGETABLE ADDED; [H0160] = ALCOHOLATED; [H0349] = ONION ADDED.

For all three products, the following descriptors, respectively, related to *preservation method*, *container/wrapping*, are used: [J0131] = PRESERVED BY CHILLING; [M0124] = PLASTIC TRAY OR PAN, PLASTIC COVER OR WRAPPING.

The products have the same type of packaging: plastic tray with plastic sealing film, indicated as [M0124] = PLASTIC TRAY OR PAN, PLASTIC COVER OR WRAPPING. In the remark section, the type of plastic to which the tray and film belong has been specified: the tray is made of polypropylene (type 05) and the plastic film is mixed plastic (type 07).

##### First Courses, Cereal Salad

According to the descriptors chosen for the *product type* facet, the following combinations of descriptors is used: -[A0822] = SAVOURY CEREAL DISH (EUROFIR); [A0106] = PREPARED GRAIN OR STARCH PRODUCT (US CFR) or -[A0861] = PREPARED FOOD PRODUCT (EUROFIR); [A0172] = PREPARED FOOD PRODUCT (US CFR).

The facet [B] descriptors are as follows: [B2917] = SPELT; [B1448] = SORGHUM; [B2027] = QUINOA; [B1047] = GRAIN OR SEED-PRODUCING PLANT; [B1405] = BUCKWHEAT.

Connected to the above-mentioned facet, [C0155] = SEED (for three products) and [C0134] = SEED, SKIN REMOVED (for two products) were used.

For the facet [E] *physical state*, *shape or form*, the descriptor [E0104] = WHOLE AND PIECES was applied to all five products.

The descriptors of the facet [F] *extent of heat treatment* used are [F0014] = FULLY HEAT-TREATED (for four products) and [F0023] = HEAT-TREATED, MULTIPLE COMPONENTS, DIFFERENT DEGREES OF TREATMENT (for one product).

The facet [G] descriptors used are: [G0021] = COOKED IN STEAM (for four products) and [G0015] = BOILED AND DRAINED (for one product).

Because cereal salads are rich in a variety of ingredients, the following facet [H] descriptors are used: [H0367] = SALT ADDED (for five items); [H0180] = FOOD ADDED (for four items); [H0341] = OLIVE OIL ADDED (for four items); [H0212] = VEGETABLE ADDED (for two items) and for one item, the following descriptors: [H0320] = CORN ADDED; [H0347] = SAFFLOWER OR SUNFLOWER OIL ADDED; [H0350] = TOMATO ADDED; [H0321] = RICE ADDED; [H0333] = SEED ADDED.

Regarding *preservation methods*, [J0004] = PRESERVED BY OTHER METHOD is used for ambient products (four products) and [J0131] = PRESERVED BY CHILLING for one product.

For describing *container/wrapping*, the following descriptors are used: [M0166] = PLASTIC BAG OR POUCH (for four items) and [M0124] = PLASTIC TRAY OR PAN, PLASTIC COVER OR WRAPPING (for one item). For [M0166] = PLASTIC BAG OR POUCH is specified in the remarks section that the pouch (bag) is made of plastic type 07 (mixed plastic); for [M0124] = PLASTIC TRAY OR PAN, PLASTIC COVER OR WRAPPING, the type of plastic tray, plastic type 05, and a sealing film to cover, plastic type 07, are specified in the remarks section.

#### 3.1.5. Sandwiches

For all five products classified as sandwiches, the two descriptors [A1203] = SANDWICH (EUROFIR); [A0218] = SANDWICH (US CFR) are used together.

For all items, the descriptor [B1421] = SOFT WHEAT and related [C0208] = SEED, SKIN REMOVED, GERM REMOVED (ENDOSPERM) were added. For [C0208] = SEED, SKIN REMOVED, GERM REMOVED (ENDOSPERM), the type of flour was specified in the remarks section: flour type “0” for one item, and type “00” for another one.

For the facet [E], [F], and [G] the same descriptors, used respectively as follows, were applied to all five products: [E0151] = SOLID; [F0003] = NOT HEAT TREATED; [G0003] = COOKING METHOD NOT APPLICABLE.

The following facet [H] descriptors are used: [H0207] = FILLED OR STUFFED (for four items); [H0765] = HAM ADDED (for three items); [H0117] = FLAVORING OR TASTE INGREDIENT ADDED (for three items); [H0153] = SEAFOOD ADDED (for two items); [H0143] = CHEESE ADDED (for two items); [H0362] = FRUIT JUICE ADDED (for one item); [H0367] = SALT ADDED (for one item). For [H0765] = HAM ADDED has been specified in remarks section of three products the type of ham: cooked ham; for [H0117] = FLAVORING OR TASTE INGREDIENT ADDED has been specified in two items that mayonnaise was added, whereas in another item, that mayonnaise and mustard were added; for [H0153] = SEAFOOD ADDED, the type of seafood added has been specified: in one item, the seafood was tuna, whereas in the other, it was salmon; for [H0362] = FRUIT JUICE ADDED, the specification refers to lemon juice added; for [H0143] = CHEESE ADDED, it has been specified that the type of cheese added was mozzarella.

The descriptor [J0131] = PRESERVED BY CHILLING is added to all items belonging to this category to indicate the *preservation method* used.

The following facet [M] descriptors were used: [M0124] = PLASTIC TRAY OR PAN, PLASTIC COVER OR WRAPPING (for four items) and [M0166] = PLASTIC BAG OR POUCH (for one item).

The descriptor [M0124] = PLASTIC TRAY OR PAN, PLASTIC COVER OR WRAPPING is used to indicate that the container is presented as a plastic tray composed of a plastic film as a sealing element. In the remarks, there are different types of plastic for these components stated. In another case, the product was inside a box, which was a package with a lid that was part of the box itself. There was no film actually present, but there was a plastic element that closed.

#### 3.1.6. Fruit and Derivatives

The following combination of [A] descriptors were used: -[A0866] = PREPARED SALAD (EUROFIR); [A0172] = PREPARED FOOD (US CFR); and -[A0841] = JUICE OR NECTAR (EUROFIR); [A0196] =FRUIT NECTAR (US CFR).

The following facet [B] descriptors were used: [B1140] = FRUIT-PRODUCING PLANT (for three items) and [B1339] = ORANGE (for one item). For [B1339] = ORANGE, the type of orange has been specified in remark field: blond oranges.

The facet [C] descriptors used are as follows: [C0167] = FRUIT (for three items) and [C0229] = FRUIT, PEEL REMOVED, CORE, PIT OR SEED REMOVED (for one item).

The facet [E] descriptors used are: [E0104] = WHOLE AND PIECES; [E0121] = LIQUID, HIGH VISCOSITY, WITH SMALL PARTICLES; [E0152] DIVIDED INTO PIECES; [E0135] = SEMILIQUID WITH SMOOTH CONSISTENCY.

The descriptors [F0003] = NOT HEAT-TREATED and [G0003] = COOKING METHOD NOT APPLICABLE are used for all four items.

[H0212] = VEGETABLE ADDED and [H0147] = FRUIT ADDED have been used. For [H0212] = VEGETABLE ADDED, it has been specified in the remarks section that carrots were added. For [H0147] = FRUIT ADDED, the type of fruits added was specified: a mix of apple, banana, lemon, and grapes, all blended together (in smoothies) and introduced alongside the primary ingredient, oranges.

The following facet [J] descriptors are used: [J0131] = PRESERVED BY CHILLING (for three items), and [J0160] = STERILIZED BY ULTRA HIGH PRESSURE (for one item).

The following facet [M] *container/wrapping* descriptors are used: [M0124] =PLASTIC TRAY OR PAN, PLASTIC COVER OR WRAPPING (for three items), [M0430] = POLYETHYLENE TEREPHTHALATE (PET) CONTAINER (for two items), [M0214] = BOTTLE (for one item), [M0237] = PLASTIC SCREW CAP OR LID (for one item). The descriptor [M0124] = PLASTIC TRAY OR PAN, PLASTIC COVER OR WRAPPING is used for three items. In the remarks section, it was clarified once that it refers to a plastic tray covered by a plastic film as a sealing element. In other instances, it is paired with [M0430] = POLYETHYLENE TEREPHTHALATE (PET) CONTAINER, which already indicates the material type, so no remarks were made about the material, since it was already stated explicitly that a box with a lid was included in the structure. Additional details were not needed, as the lid is part of the tray structure and is also made of PET. For [M0214] = BOTTLE, it was specified that the bottle is made of PET material. Likewise, for [M0237] = PLASTIC SCREW CAP OR LID, it was specified that the cap is made of HDPE-2, the specific plastic material.

### 3.2. Application of FoodEx2

Table S3 (see Supplementary Materials) reports the FoodEx2 codes of the 50 products. As already anticipated for FoodEx2, returning a shorter code presents implicit information inside it, which from the code can be traced. In the *Hierarchy* drop-down menu, the *Exposure Hierarchy* list was chosen to address the coding work that is supported by adding facets. The facets are 32 in total. The descriptors used for coding the fifty ready-to-eat products belong to the following facet: F.28 *process*; F.04 *ingredient*; F.18 *packaging*—*format*; F.19 *packaging*—*material*.

The approach adopted in this research to insert ingredients using a code was to add only the most representative ones, with a maximum of five presences per product when it was possible and satisfactory. The thought approach, including the choice of limited facets, has been designed to minimize the length of the resulting code. Being composite dishes, a complex matrix is coded, rich in added ingredients and with high presence of other elements.

#### 3.2.1. Salads

Almost all classified as salads have been codified by the *base term*, *Mixed green salad* [A042C]. Only for one product was the base term *Greek salad* [A042F] used. Instances of the coding procedure for product (item 11—*Mixed green salad*) are: *Escaroles* [F04.A00LE], *Roman rocket* [F04.A00LN], *Cheese*, *grana padano* [F04.A02ZQ], *Walnuts* [F04.A014R], *Cherry tomatoes* [F04.A00HY]; as process: *Chilling* [F28.A07KP]; as *packaging—format*: *Tray* [F18.A0F2D] and *packaging—material*: *Polyethylene Terephthalate (PET, PETE)* [F19.A16RP], *Plastic* [F19.A07PR] (see Supplementary Materials).

The resulting code string will display A042C#F28.A07KP$F04.A00LE$F04.A00LN$F04.A02ZQ$F04.A014R$F04.A00HY$F18.A0F2D$F19.A16RP$F19.A07PR.

The code always starts with the *base term*, which is in our example *Mixed green salad* with *term code* [A042C], followed by a hash (#) to separate the *base term* from the next facet; this can be seen in the example A042C#F28.A07KP$, where F28 is the facet related to the process and [A07KP] is the *term code* equivalent to *Chilling*. Facets are separated by the dollar sign ($). At the end of the code, there are no symbols. After the process descriptors, the ingredients, facet F04, appear. All the facets are separated by a dollar sign, always. Follow F18 (packaging—format) and F19 (packaging—material) descriptors. “F04”, “F28” as well as “F18” and “F19“, are followed by a point (.) separating them from the corresponding *term code*.

Words such as *Chilling*, *Escaroles*, or, for example, *Plastic*, are called the *term name*. The corresponding alphanumeric code is the *Term code*.

F28 (process), F04 (ingredients), F18 (packaging—format), and F19 (packaging—material) are called *Facets*. The alphanumeric code at the top of the code is called the *base term*, which corresponds to the question “WHAT TYPE OF FOOD IS THIS?”.

For all items, *Chilling* [F28.A07KP] (under *F.28 process*), describes storage at refrigeration temperature (keeping the product at a temperature higher than that relative to the freezing point).

For the facet *F.04 ingredient*, the following descriptors are used for coding all items under *Salads*: *Escaroles* [F04.A00LE] (for three item); *Roman rocket* [F04.A00LN] (for three items); *Cherry tomatoes* [F04.A00HY] (for two items); *Table olives ready for consumption* [F04.A01BQ] (for two items); *Radicchio* [F04.A00LG] (two times); *Sweet corn* [F04.A00KP] and for one item: *Carrots* [F04.A00QH]; *Cheese*, *grana padano* [F04.A02ZQ]; *Walnuts* [F04.A014R]; *Lamb’s lettuces* [F04.A00KT]; *Extra hard cheese* (parmesan, grana type) [F04.A02ZH].

For the facet *F.18 packaging—format*, two descriptors were used: *Tray* [F18.A0F2D] (for four items) and *Bag*, *sack*, *or pouch* [F18.A07NK] (for one item).

For the facet *F.19 packaging—material*, three descriptors were used to describe the plastic material of the product packaging: *Polyethylene Terephthalate (PET, PETE)* [F19.A16RP] (for three items); *Plastic* [F19.A07PR] (for two items); *Polypropylene (PP)* [F19.A16RX] (for one item).

Additional comments and specific details were inserted in the remarks field. As instance, for the facet *F.04 ingredient*, details were added for *Table olives ready for consumption* [F04.A01BQ]: for one product, to specify that table olives were black and pitted green olives, and for the other one, to specify that table olives were pitted green olives alone.

Additional remarks were used to explain the type of plastic material of the packaging. The following are examples of the products concerned. The product that presents as applied descriptors: PACKAGING—FORMAT = *Bag*, *sack*, *or pouch* [F18.A07NK]; PACKAGING—FORMAT = *Tray* [F18.A0F2D]; PACKAGING—MATERIAL = *Polyethylene Terephthalate (PET, PETE)* [F19.A16RP], PACKAGING—MATERIAL = *Polypropylene (PP)* [F19.A16RX] saw the use of following remark: the material indicated as PET is related to *Bag*, *sack*, *or pouch*, while the material indicated as polypropylene is related to the tray.

For another product, presenting as packaging descriptors applied: PACKAGING—FORMAT = *Tray* [F18.A0F2D], PACKAGING—MATERIAL = *Plastic* [F19.A07PR], this part of the code will appear as follows [...$F18.A0F2D$F19.A07PR…]. PACKAGING—MATERIAL = *Plastic* has been chosen because the category of plastic has not been made explicit, and in the remark, it was mentioned that it corresponds to both the PACKAGING—FORMAT = *Tray* but also to the plastic film that is present as the sealing element of the tray. Since the specification of the plastic category used is not present, the descriptor PACKAGING—MATERIAL = Plastic was considered relevant. The other two products have seen explicitly that the PET material is related to the tray and that the plastic material refers instead to the tray film as a sealing element.

#### 3.2.2. Side Dishes

The side dishes category counts a total of nineteen products, classified by *base term* as follows: *Vegetable-based dishes* [A03XX] have been selected for fourteen products, and *Potato-based dishes* [A03VD] for two products. The other three are classified as follows: *Vegetables*, *gratinated* [A03YF]; *Mixed vegetables*, *boiled* [A03YE]; *Mixed vegetables* [A16GB].

The descriptor *Chilling* [F28.A07KP] is applied for all items. Seven of these products are stored under vacuum, the chosen descriptor is *Vacuum-packing* [F28.A07JK]. The *Boiling* [F28.A07GL] and *Steaming* [F28.A07GP] process descriptors were used for five items. Also, the following descriptors are used: *Pan Frying/Shallow Frying* [F28.A07GS], *Slicing* [F28.A07KV], *Broiling/grilling* [F28.A07GZ], *Baking* [F28.A07GX], *Battering* [F28.A07HL], *Dicing and stripe-cutting* [F28.A07KX]. Moreover, for *Boiling* [F28.A07GL], it has been specified in the remarks section that draining occurred. For *Broiling/grilling* [F28.A07GZ], it has been specified that it is related to the grilling process.

The descriptors under facet [F04] are used for describing ingredients and specifies added in the remarks section.

For the packaging—format, the descriptor *Tray* [F18.A0F2D] was used for twelve items, and the descriptor *Bag*, *sack*, *or pouch* [F28.A07NK] for seven items.

For the *packaging*—*material*, the descriptor *Plastic* [F19.A07PR] was used for nineteen items, and the descriptor *Polypropylene (PP)* [F19.A16RX] for eight items. Eight times, we find the combination: PACKAGING—MATERIAL = *Polypropylene (PP)* [F19.A16RX], PACKAGING—MATERIAL = *Plastic* [F19.A07PR], PACKAGING—FORMAT = Tray [F18.A0F2D]. Moreover, in the remarks, it has been specified that the polypropylene material refers to *tray* (tray, facet F18 belonging to the *packaging*—*format*), while the material “plastic” is referred to the tray’s film, intended as a sealing element. The film is type 07, composed of mixed plastic identified under the code “07”. For the other seven items, the following combination of descriptors is applied: descriptor PACKAGING—FORMAT = *Bag*, *sack*, *or pouch* [F18.A07NK] and PACKAGING—MATERIAL = Plastic [F19.A07PR]. It is pointed out in the remarks that it was type 07 plastic, but in these cases in the code, we have it that the *format* is the bag. In four cases, we have it that the plastic material [F19.A07PR] belongs to both the tray [F18.A0F2D] and its film used as a sealing element; this is because the type of plastic is unknown to us. In two cases, remarks were not made, and this is explained as follows: the plastic descriptor satisfied the coding work because the type of plastic is unknown to us, and having only one format in the code, the correlation is evident.

#### 3.2.3. Second Courses

A total of eight products are within the *Second courses* category: five products are fish-based dishes, of which four are classified with base term *Fish and seafood-based dishes* [A03XJ], and one with the base term *Prepared fish salad* [A03XP]. The remaining three products under *Second courses* are meat-based dishes and are classified with base term *Meat-based dishes* [A03VV].

For all items under *Second courses*, the descriptor inherent to the facet of the *Process*, *Chilling* [F28.A07KP] is used. Being that six of these products are sliced, the descriptor *Slicing* [F28.A07KV] is used. Four of these products are marinated, one of which is smoked, so *Marinating* [F28.A07JT] and *Smoking* [F28.A07JV] were chosen as descriptors. The descriptors listed below were also used: *Broiling/grilling* [F28.A07GZ], *Deep Frying* [F28.A07GV], *Steaming* [F28.A07GP], *Breading* [F28.A07HK], *Baking* [F28.A07GX]. Moreover, for the descriptor *Baking* [F28.A07GX] has been specified in the remarks section that this process took place at low temperatures.

For indicating ingredients for the products under *Second courses*, descriptors under facet F04 *ingredients* are used.

For *packaging*—*format*, the descriptors *Tray* [F18.A0F2D] (for seven items) and *Bag*, *sack*, *or pouch* [F18.A07NK] (for one item) are used. For *packaging*—*material*, the following descriptors were used: *Plastic* [F19.A07PR] (for six item), *Polyethylene Terephthalate (PET, PETE)* [F19.A16RP] (for one item), *Polypropylene (PP)* [F19.A16RX] (for one item); *Polyvinyl Chloride (PVC)* [F19.A16RT] (for one item). The remaining remarks concern the correlation between the *packaging*—*material* and its *format*.

#### 3.2.4. First Courses

For the *First courses* the following sub-categories: *Pasta*, *Soups*, and *Cereal salad*.

##### First Courses, Pasta

There are two products in this category. The *base term* used for the two items is *Pasta-based dishes*, *cooked* [A040N].

*F.28* is the facet inherent in the *process*. For one product the following process descriptors are used: PROCESS = *Boiling* [F28.A07GL], PROCESS = *Chilling* [F28.A07KP]. The other product present an additional process, and the following process descriptors are used: PROCESS = *Boiling* [F28.A07GL], PROCESS = *Pan Frying/shallow frying* [F28.A07GS], PROCESS = *Chilling* [F28.A07KP]. Moreover, for *Boiling* [F28.A07GL], it has been specified that draining occurred.

*F.04* is the facet inherent in the *ingredients*. For one product, the descriptors of the main ingredients used are as follows: INGREDIENT = *French beans (with pods)* [F04.A00PG], INGREDIENT = *Potatoes* [F04.A00ZT], INGREDIENT = *Pesto* [F04.A044V], INGREDIENT = *Olive oil*, *virgin or extra virgin* [F04.A036Q]. For the other product, the descriptors are as follows: INGREDIENT = *Broccoli* [F04.A00FN], INGREDIENT = *Italian-style sausage* [F04.A024H], INGREDIENT = *Olive oil*, *virgin or extra virgin* [F04.A036Q]. The two products share extra-virgin olive oil as an ingredient. The other ingredients are different. Moreover, for *Pesto* [F04.A044V] indicated as an added ingredient, the composition of the same has been explained in the remark, below: sunflower seed oil, basil, Grana Padano PDO. Moreover, for *Olive oil*, *virgin or extra-virgin* [F04.A036Q], the type of oil: extra-virgin type, has been specified.

The descriptor PACKAGING—FORMAT = *Tray* [F18.A0F2D] is used for both products. In the remarks, it has been specified that a film is used as a sealing element. For both products, the following two descriptors for packaging—materials, *Polypropylene (PP)* [F19.A16RX] and *Plastic* [F19.A07PR], are applied, and in the remarks section, it has been specified that *Polypropylene* is the material of the tray, and the *plastic* material refers to the film of tray, intended as a sealing element.

##### First Courses, Soups

For one product, the base term *Mixed vegetables soup* [A041S] has been used, for another one, *Mixed soups* [A0CDN], and for the last one, *Legume-based dish* [A03VM] was used.

The descriptors *Chilling* [F28.A07KP] and *Simmering* [F28.A07GJ] were applied to all three products.

For the product classified as *Mixed vegetables soup* [A041S], the following descriptors for ingredients are added: *Courgettes* [F04.A00JR], *Carrots* [F04.A00QH], *Spinach* [F04.A00MJ], *Leeks* [F04.A00SB], *Onions* [F04.A00HC], *Potatoes* [F04.A00ZT], *Celery leaves* [F04.A00XA], *Savoy cabbages* [F04.A00GB], *Garden peas (without pods)* [F04.A012J], = *Olive oil*, *virgin or extra virgin* [F04.A036Q]. Moreover, for *Olive oil*, *virgin or extra virgin* [F04.A036Q], it has been specified that the type of oil is extra-virgin olive oil. For the products classified as *Mixed soups*, the following descriptors for ingredients are added: *Courgettes* [F04.A00JR], *Carrots* [F04.A00QH], *Leeks* [F04.A00SB], *Celery leaves* [F04.A00XA], *Barley grains* [F04.A000P], *Spelt grain* [F04.A001R], *Borlotti or other common beans (without pods)* [F04.A012B], *Tomato puree* [F04.A00ZD], *Aromatic herbs* [F04.A04MA]. For the product classified as *Legumes based dish*, the following descriptors for ingredients are added: *Lentils (without pods)* [F04.A012L], *Olive oil*, *virgin or extra-virgin* [F04.A036Q], *Garlic* [F04.A00GZ], *Onions* [F04.A00HC], *Carrots* [F04.A00QH], INGREDIENT = *Wine* [F04.A03MT].

For two products, the packaging—format and packaging—material are indicated as: PACKAGING—FORMAT = *Tray* [F18.A0F2D]; PACKAGING—MATERIAL = *Polypropylene (PP)* [F19.A16RX]; PACKAGING—MATERIAL = *Plastic* [F19.A07PR]. The PACKAGING—MATERIAL indicated as Polypropylene (PP) refers to the Tray. The PACKAGING—MATERIAL indicated as plastic is refers to the tray’s sealing film (type 07). For the other one, the descriptors are as follows: PACKAGING—FORMAT = *Tray* [F19.A0F2D], PACKAGING—MATERIAL = *Plastic* [F19.A07PR]. The third product, presented as plastic [F19.A07PR] material, commented on the fact that they were bioplastics, such as Mater-Bi for the tray and PLA for the sealing film. Having been used in the various remarks of this work, the descriptor plastic was used to describe and classify those products in mixed plastic (type 07) or unknown plastic (because the manufacturer did not report it) types, and bioplastics also belonged to this category, which is the descriptor that has been chosen for the classification.

##### First Courses, Cereal Salads

The *base term Cereal-based dishes (other than pasta and rice)* [A18PS] was used for classifying items under this category because it takes into account cereals, but excludes pasta and rice, which are not present in this product as main ingredients.

The descriptor *Steaming* [F28.A07GP] is used to indicate the cooking method of four products; for the other product, *Boiling* [F28.A07GL] is used for indicating the cooking method and *Chilling* [F28.A07KP] for indicating storage at a refrigeration temperature. Note that products subjected to steaming do not include storage at refrigeration temperature. They are in fact products belonging to the “ambient” category. Moreover, in the remark section for *Boiling* [F28.A07GL], it is indicated that draining occurred.

The descriptors under Facet F04 are used for indicating ingredients and details specified in remarks.

For *packaging*—*format*, the descriptor *Bag*, *sack*, *or pouch* [F18.A07NK] is applied for four products; for another product packaged in a tray, the descriptor *Tray* [F18.A0F2D] is applied. For *packaging*—*material*, the descriptor *Plastic* [F19.A07PR] is applied for four products and the descriptor *Polypropylene (PP)* [F19.A16RX] for one product. Details on *packaging*—*format* and *packaging*—*material* are added in the remarks section.

#### 3.2.5. Sandwiches

The base terms used for classifying the items under Sandwiches are: *Sandwich with processed meat topping/filling* [A03ZB]; *Sandwich with fish topping/filling* [A03ZC]; *Sandwich and sandwich-like dishes* [A03YZ]

The descriptor PROCESS = *Chilling* [F28.A07KP] is used for all products. The descriptors used for indicating the type of bread used as a main ingredient are as follows: INGREDIENT = *Wheat bread and rolls* [F04.A004X] for four products, and INGREDIENT = *Unleavened or flat bread and similar* [F04.A04KZ] for one product that is made of a flat, thin bread. Descriptors used for indicating other ingredients are as follows: *Cooked pork ham* [F04.A023K] (for three items), *Mayonnaise sauce* [F04.A044X] (for three items), *Tuna* [F04.A02DX] (for one item); *Cheese*, *edam* [F04.A02TV] (for one item); *Smoked salmon* [F04.A02KF] (for one item), and *Mozzarella* [F04.A02QJ] (for one item).

For *packaging*—*format *, the descriptor *Tray* [F18.A0F2D] is used for four products, and the descriptor *Bag*, *sack*, *or pouch* [F18.A07NK] is used for one product, whereas for *packaging—material*, the descriptor *Plastic* [F19.A07PR] is used for all products. Comments are added in the remarks for the four products to specify the type of plastic.

#### 3.2.6. Fruit and Derivatives

In the last category, we find the one related to fruit and its derivatives. Out of a total of four products, two are fruit salads, classified by the base term *Fruit salad* [A01QG]. The other two products are, respectively, *Fruit smoothies* [A03DF] and *Fruit or fruit*—*vegetable puree* [A01QJ].

The descriptor *Chilling* [F28.A07KP] is applied to all items. All categories require a cold chain as a method of preservation. For one product, the descriptor *Dicing and stripe-cutting* [F28.A07KX] is also added to indicate the cutting process in the form of cubes; for another product, the descriptor *High-pressure treating (Pascalization)* [F28.A07JC] is used to indicate that this product has undergone Pascalization as a preservation process.

Descriptors used for ingredients are: *Apples* [F04.A01DJ], *Table grapes* [F04.A01DX]; *Pineapples* [F04.A01LP], *Common banana* [F04.A01LC], *Kiwi fruits* (*green*, *red*, *yellow*) [F04.A01JT]; *Juice*, *orange* [F04.A03AM]; *Carrots* [F04.A00QH]; *Lemons* [F04.A01BY]; *Mangoes* [F04.A01LF]; *Strawberries* [F04.A01EA]. Moreover, some specifics are added in the remarks section.

For *packaging*—*format*, the descriptor *Tray* [F18.A0F2D] is used for three products, whereas, for the another one, *Bottle* [F04.A07NM] is used. For *packaging—material* the following descriptors are used: *Polyethylene Terephthalate (PET, PETE)* [F19.A16RP] (for three products), *High-Density Polyethylene (HDPE)* [F19.A16RR], and *Plastic* [F19.A07PR] (for one product), and specifics are added in the remarks section.

## 4. Conclusions

A database of LanguaL^TM^ and FoodEx2 codes of 50 ready-to-eat products was developed. Both LanguaL^TM^ and FoodEx2 systems are well suited to the work of classifying and describing foods and have therefore fully satisfied these aspects in their application to include ready meals designed for the lunch break, in an environment that is both work and home.

The codes produced for the ready-to-eat products in this study make these products suitable to be inserted in Food Databases, allowing an easy exchange from the databases of more countries, going beyond the limit imposed by the language barrier, a limit which is overcome thanks to the support of coding software such as LanguaL^TM^ and FoodEx2.

The database of LanguaL^TM^ and FoodEx2 codes of 50 ready-to-eat products was developed and reported in the Supplementary results as a tool for data standardization and data sharing in several potential applications, such as nutrional cards, nutritional facts, and food labels or booklet and brochures for promotion of food products, at interfaces of health and food nutrition, useful for several stakeholders and beneficiaries, i.e., consumers, nutritionists, dieticians, and food producers/processors. In this way, the data exchange occurs quickly and consistently.

In the coding work carried out by applying LanguaL^TM^ and FoodEx2 to meals ready for consumption outside the home, it is important to evaluate the aspect relating to packaging. The descriptors that help in this work are those that belong to the [M] facet, relating to container or wrapping, for the LanguaL^TM^ system. In coding through FoodEx2, this aspect is evaluated by two distinct but related facets such as F18 packaging (format) and F19 packaging (material).

The future challenge is the application of coding systems at different levels of the food supply chain, from farm to fork, i.e., agriculture, nutrition, food manufacturing, distribution, allowing for people to discriminate between different products based on peculiar characteristics linked to the environment, production, process technology, etc.

## Data Availability

The authors declare that the data supporting the findings of this study are available within the article. All the other data are available on reasonable request from the corresponding authors.

## Supplementary Materials

The following supporting information can be downloaded at: https://www.mdpi.com/article/10.3390/nu16081151/s1, Supplementary material consists of: Table S1: Ingredients, treatments, processing, and cooking method applied, storage/preservation method, info packaging (format and material), of the 50 products; Table S2: LanguaL^TM^ codes of the 50 products; Table S3: FoodEx2 codes of the 50 products.
